# How Does Cardiovascular Disease First Present in Women and Men? Incidence of 12 Cardiovascular Diseases in a Contemporary Cohort of 1 937 360 People

**DOI:** 10.1161/CIRCULATIONAHA.114.013797

**Published:** 2015-10-05

**Authors:** Julie George, Eleni Rapsomaniki, Mar Pujades-Rodriguez, Anoop Dinesh Shah, Spiros Denaxas, Emily Herrett, Liam Smeeth, Adam Timmis, Harry Hemingway

**Affiliations:** From Farr Institute of Health Informatics Research (London), University College London, United Kingdom (J.G., M.P.-R., A.D.S., S.D., H.H.); Worldwide Clinical Trials, Nottingham, United Kingdom (E.R.); Farr Institute of Health Informatics Research (London), London School of Hygiene & Tropical Medicine, United Kingdom (E.H., L.S.); and Farr Institute of Health Informatics Research (London) and Barts National Institute for Health Research Cardiovascular Biomedical Research Unit, Queen Mary University of London, United Kingdom (A.T.).

**Keywords:** aging, cardiovascular diseases, electronic health records, incidence, population, risk factors, sex

## Abstract

Given the recent declines in heart attack and stroke incidence, it is unclear how women and men differ in first lifetime presentations of cardiovascular diseases (CVDs). We compared the incidence of 12 cardiac, cerebrovascular, and peripheral vascular diseases in women and men at different ages.

**Methods and Results—:**

We studied 1 937 360 people, aged ≥30 years and free from diagnosed CVD at baseline (51% women), using linked electronic health records covering primary care, hospital admissions, acute coronary syndrome registry, and mortality (Cardiovascular Research Using LInked Bespoke Studies and Electronic Records [CALIBER] research platform). During 6 years median follow-up between 1997 and 2010, 114 859 people experienced an incident cardiovascular diagnosis, the majority (66%) of which were neither myocardial infarction nor ischemic stroke. Associations of male sex with initial diagnoses of CVD, however, varied from strong (age-adjusted hazard ratios, 3.6–5.0) for abdominal aortic aneurysm, myocardial infarction, and unheralded coronary death (particularly >60 years), through modest (hazard ratio, 1.5–2.0) for stable angina, ischemic stroke, peripheral arterial disease, heart failure, and cardiac arrest, to weak (hazard ratio <1.5) for transient ischemic attack, intracerebral hemorrhage, and unstable angina, and inverse (0.69) for subarachnoid hemorrhage (all *P*<0.001).

**Conclusions—:**

The majority of initial presentations of CVD are neither myocardial infarction nor ischemic stroke, yet most primary prevention studies focus on these presentations. Sex has differing associations with different CVDs, with implications for risk prediction and management strategies.

**Clinical Trial Registration—:**

URL: http://www.clinicaltrials.gov. Unique identifier: NCT01164371.

## Abstract 2

Supplemental Digital Content is available in the text.

A central principle in cardiovascular disease (CVD) management is that the first lifetime diagnosis signals the failure of primary prevention and the need to initiate secondary prevention of recurrent or related CVD events. The decades-long emphasis given to prevention of myocardial infarction (MI) and stroke is reflected in remarkable declines – ≈33% over the past decade – in their incidence in developed countries.^1^ Incidence rates for chronic CVD presentations such as angina or heart failure, although less studied, do not appear to have similarly declined.^1–3^ Consequently, the spectrum of initial presentations of CVD in contemporary practice is likely to have changed in comparison with the latter part of the last century. Cohort studies that report only fatal end points (final presentations),^4^ may have less relevance to informing the success of primary prevention than those which investigate initial presentations. Within studies that incorporate nonfatal events, acute MI and stroke have been more commonly investigated than other chronic presentations.^5–7^ Large-scale contemporary studies that evaluate the first lifetime diagnosis in women and men across a wide range of acute and chronic CVDs including both fatal and nonfatal presentations can provide additional insight into the understanding of CVDs.

**Editorial see p 1303**

**Clinical Perspective on p 1328**

Fundamental unanswered questions about initial CVD presentation arise. First, what is the relative frequency of different CVDs as they affect women and men in contemporary practice? Second, is male sex an equally strong risk factor common to all CVDs, or does the association differ across a range of diseases?

The lack of large, contemporary, population-based cohorts with detailed clinical follow-up spanning hospital and ambulatory care has hindered the study of the initial presentation of a wide range of acute and chronic CVDs. It has been suggested that electronic health record (EHR) data might be meaningfully reused^8^ to create mega-cohorts for such research.^9^ We studied a contemporary, population-based cohort based on linked EHRs across primary, secondary, disease registry, and death records^10–13^ to address these 2 questions. We investigated a wide range of acquired symptomatic CVDs that are recognized to have differing pathogenic mechanisms.

## Methods

### Data Sources

Anonymized patients were selected from the Cardiovascular Research Using LInked Bespoke Studies and Electronic Records (CALIBER) program, described^14^ and validated^10–13,15^ elsewhere. Patients were linked across 4 clinical data sources: the Clinical Practice Research Database (CPRD), the Myocardial Ischemia National Audit Project registry, Hospital Episodes Statistics, and the national death registry from the Office for National Statistics. CPRD provides primary care data on anthropometric measurements, laboratory tests, medical history, clinical diagnoses, prescriptions, medical procedures, and health behaviors, coded using the Read clinical coding scheme. Patients registered in practices submitting linkable data to CPRD, covering ≈4% of the English population, have been found to be representative of the English population in terms of age, sex, and ethnicity.^16,17^ Myocardial Ischemia National Audit Project is a national registry of patients admitted to the hospital with acute coronary syndromes. Hospital Episodes Statistics provides information on diagnoses and medical procedures related to all elective and emergency hospital admissions across all National Health Service hospitals in England.

### Study Population

We studied 1 937 360 patients from 225 general practices across England registered between January 1997 and March 2010. We required that at study entry patients were aged ≥30 years, were free of diagnosed CVD, and had been followed up for at least 1 year. We used the entire medical history available on each patient to confirm they were free of diagnosed CVD. The look-back period ranged from 20 years to the minimum of 1 year, which previous research has indicated is a sufficient period to ensure accurate assessment of initial disease presentations.^18^ We used an open cohort design, so patients effectively entered the study when they met the inclusion criteria. Patients were censored on the earliest date from among: the date of first CVD presentation, date of death from other causes, date leaving the practice, or date of last practice data collection. (See Figure I in the online-only Data Supplement for study flow diagram.)

### Risk Factors

The exposures of interest were sex and baseline age, analyzed as 10-year age groups between 30 and 80. A priori confounders were baseline age as a continuous variable (in analyses estimating associations with sex), smoking status, body mass index, systolic blood pressure, total and high-density lipoprotein cholesterol, diabetes mellitus, socioeconomic status (based on area deprivation measure), use of statins, use of blood pressure medication, and, in women only, use of oral contraceptives or hormone replacement therapy. The baseline value for these confounders was taken as the most recent measurement as recorded during consultations in primary care (CPRD) up to 1 year before study entry. (Detailed definitions are in online-only Data Supplement Methods I.)

### End Points

Primary end points were defined as the first recorded diagnosis of the 12 most common symptomatic manifestations of CVD, irrespective of underlying disease mechanism, arising from pathology in the head, heart, abdomen, or legs. The first diagnosis could occur in primary care, secondary care, or at death. We studied the following CVDs: stable angina, unstable angina, nonfatal MI, unheralded coronary death (UCD), heart failure, a composite of cardiac arrest, ventricular arrhythmia, and sudden cardiac death (SCD), transient ischemic attack, ischemic stroke, subarachnoid hemorrhage (SAH), intracerebral hemorrhage, abdominal aortic aneurysm (AAA), peripheral arterial disease (PAD), composite CVD, and other deaths. In secondary analysis, we examined associations in a subset of nonfatal MIs that were classified into ST-segment–elevation MI and non–ST-segment–elevation MI. Coronary heart disease (CHD) and stroke that were not otherwise specified (NOS) were also studied. We classified as fatal events where a death record exists for the same calendar date. (Overview of codes and data sources used to define cardiovascular end points available in online-only Data Supplement Methods II.)

### Statistical Analysis

Hazard ratios (HRs) were estimated for the disease-specific Cox proportional-hazards models with length of follow-up as the timescale, stratified by practice, with women as the reference category, and included interactions between age (linear and quadratic term) and sex. Where we estimated the HR for baseline age, we additionally stratified by sex, to allow the baseline hazard to vary. The proportional hazard assumption was tested using Schoenfield residuals, with no significant effects found.

In the main analyses, we estimated the association of each end point with age groups, the age-adjusted association with sex across all subjects, and by age group in a model with sex interactions. Assuming mutual independence between initial presentations, we assessed heterogeneity in the reported associations based on τ^2^, an estimate of the between-group variance of the log hazard ratio, and a way of summarizing the variability in effect sizes across all the end points in a single statistic.^19^

In secondary analysis, we examined whether associations with sex change after adjusting for smoking status, body mass index, diabetes mellitus, systolic blood pressure, total cholesterol, high-density lipoprotein cholesterol, and social deprivation, or additionally for baseline use of blood pressure–lowering medications (diazides, β-blockers, angiotensin-converting enzyme inhibitors, angiotensin receptor blockers, or calcium channel blockers), statins, oral contraceptives and hormone replacement therapy. Missing covariate data were handled by multiple imputation. (Methods used for multiple imputation are described in online-only Data Supplement Methods III). In sensitivity analyses we studied associations between sex and CVDs (1) ignoring primary care diagnoses and (2) restricting end points to fatal events.

In a post hoc analysis, we assessed the discrimination of age- and sex-adjusted models for each of the 12 end points by calculating the separate concordance index (C-index) for each.^20^

Approval was granted by the Independent Scientific Advisory Committee of the Medicines and Healthcare Products Regulatory Agency and the Myocardial Ischemia National Audit Project Academic Group. We registered the protocol at clinicaltrials.gov (NCT01164371).

## Results

Baseline characteristics of the cohort are shown in the Table. The cohort was young at baseline, as would be expected from a population free from CVD, and 90% were white. Both systolic and diastolic blood pressure increased with age, as did the proportion on blood pressure–lowering medication, with more women than men treated at all ages. More men than women were current or ex-smokers, the proportion of current smokers declining at >60 years of age. Rates of statin prescription were low, but were higher in men than in women at all ages.

**Table.**
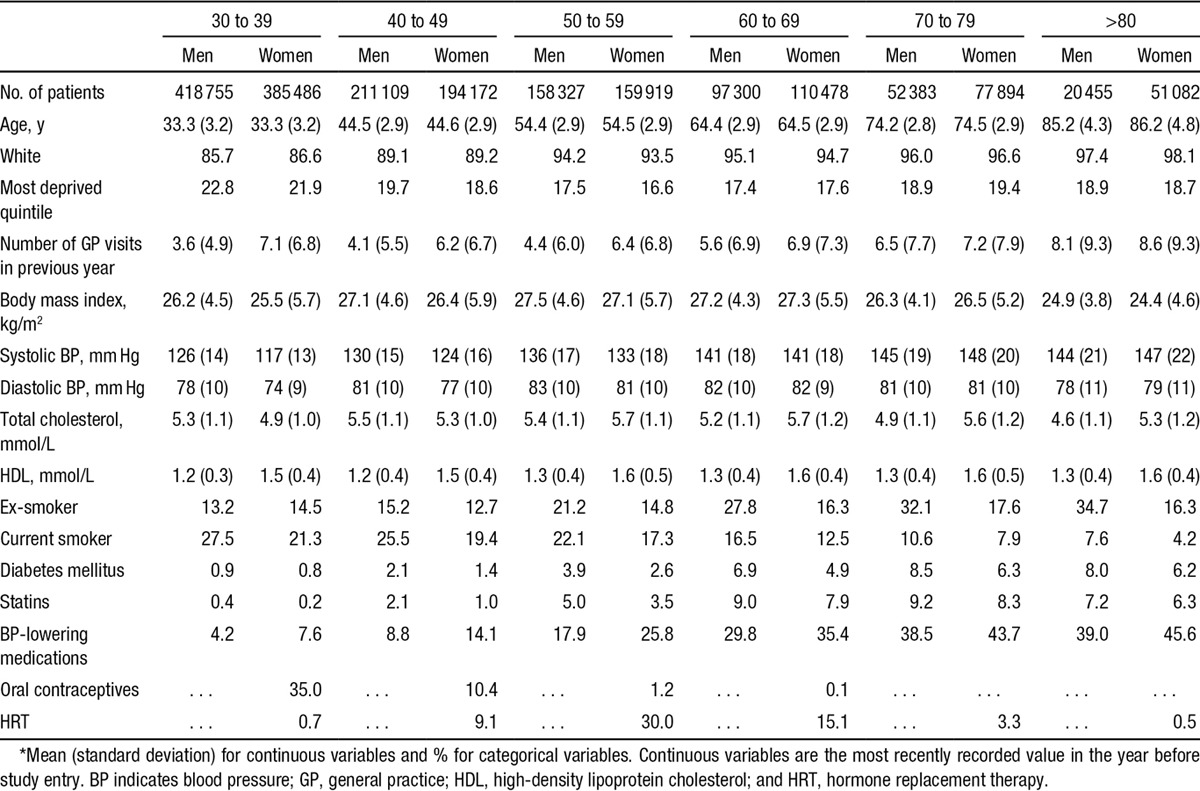
Baseline Characteristics in Men and Women by 10-Year Age Groups

### Initial CVD Presentations

**Figure 1.**
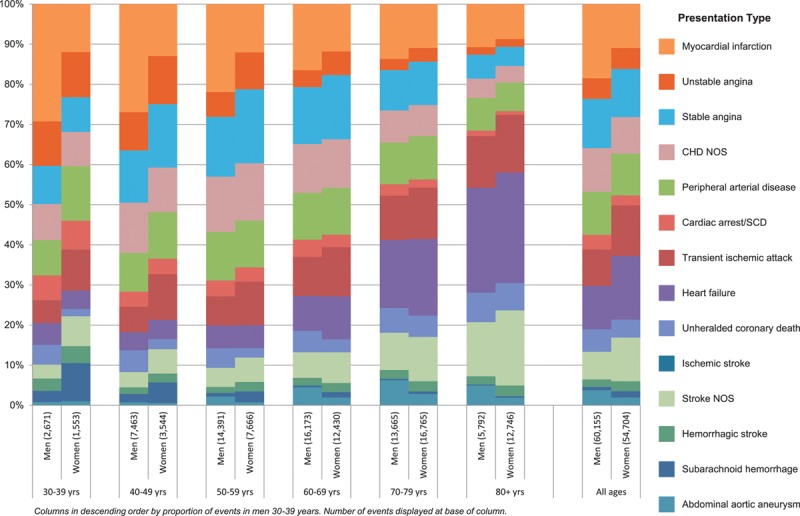
Age and sex distribution of 60 155 events in men and 54 704 in women representing the initial presentation of a wide range of CVDs. CHD indicates coronary heart disease; CVD, cardiovascular disease; NOS, not otherwise specified; and SCD, sudden cardiac death.

Over a 6-year median follow-up (interquartile range, 2–10), 114 859 initial CVD presentations were observed (52.3% in men), among which nonfatal MI, UCD, and ischemic or NOS stroke together accounted for 32.5%. The proportion of events varied by sex and age group (Figure 1; Table I in the online-only Data Supplement). The most frequent initial CVD presentation for men was nonfatal MI, which accounted for 27.9% of events in the 30 to 39 age group and more than double the proportion in women in the same age group (11.2%). This proportion declined in men as age increased, becoming similar to that in women in the >80 age group. In contrast, stable angina and unstable angina accounted for similar proportions of initial presentations in both men and women and declined with age. Although evident in younger age groups, heart failure and ischemic stroke as an initial presentation started to increase in both sexes at age 60 to form the 2 most common initial presentations at age >80.

### Associations With Age

The strength and shape of the association of CVDs with age varied from predominantly linear (in angina and nonfatal MI) to strongly quadratic (UCD, stroke, AAA), and from weak (SAH, unstable angina, and cardiac arrest/SCD) to very strong (heart failure and AAA. (See Figure II in the online-only Data Supplement.)

### Associations With Sex

**Figure 2.**
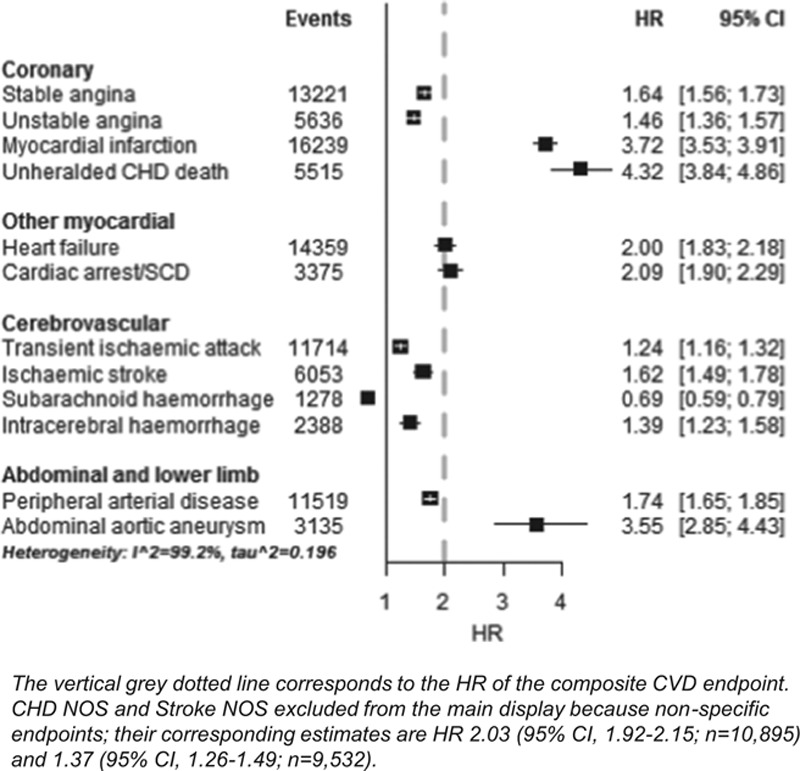
Hazard ratios of men in comparison with women for initial presentation of 12 different cardiovascular diseases among a population of 1.93 million adults. CHD indicates coronary heart disease; CI, confidence interval; CVD, cardiovascular disease; HR, hazard ratio; NOS, not otherwise specified; and SCD, sudden cardiac death.

SAH was less common in men (HR men versus women, 0.69; 95% confidence interval [CI], 0.59–0.79); other CVDs were positively associated with male sex but with considerable heterogeneity (τ^2^=0.196; Figure 2). Specifically, the age-adjusted HR (all *P*<0.001) was <1.5 for transient ischemic attack, intracerebral hemorrhage, and unstable angina, 1.5 to 2.0 for stable angina, ischemic stroke, PAD, heart failure, and cardiac arrest/SCD, and 3.6 to 5.0 for AAA, MI, and UCD. The age-adjusted HR for men versus women was 4.14 (95% CI, 3.72–4.60) in ST-segment–elevation MI and 3.18 (95% CI, 2.86–3.52) in non–ST-segment–elevation MI. These associations changed little after adjustment for conventional CVD risk factors and baseline medications, with the exception of intracerebral hemorrhage, where the association reduced to null (Figure III in the online-only Data Supplement).

**Figure 3.**
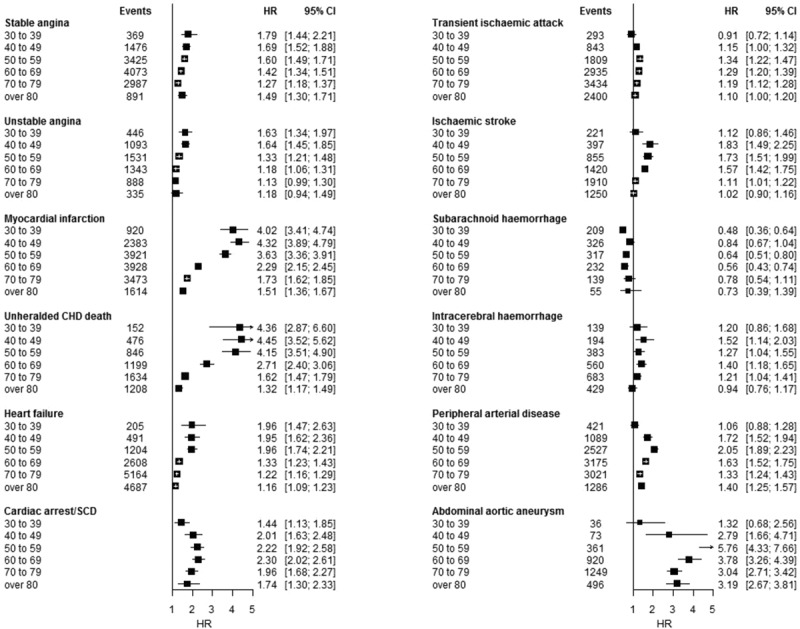
Hazard ratios for men in comparison with women for initial presentation of 12 cardiovascular diseases by baseline age group among a population of 1.93 million adults. CHD indicates coronary heart disease; CI, confidence interval; HR, hazard ratio; NOS, not otherwise specified; and SCD, sudden cardiac death.

Associations between sex and initial CVD presentation were differentially modified by age (Figure 3). The largest differences in HRs for men versus women were observed in the younger (coronary end points) and middle (ischemic stroke, PAD, AAA) age groups. Most dramatically, men <60 years old had an >4-fold higher risk of MI or UCD than similarly aged women. In all cases, associations with male sex diminished with age.

### Sensitivity Analyses

The pattern and magnitude of associations with sex were similar in multiply-adjusted analyses to analyses adjusted for age alone (see Figure III in the online-only Data Supplement). Stable angina and PAD were the only initial presentations where the association with male sex differed when the EHRs used were restricted to secondary care and mortality (Figure IV in the online-only Data Supplement).

### Discrimination of Age- and Sex-Adjusted Models for Different CVDs

Using disease-specific age and sex coefficients in risk prediction models resulted in markedly different discrimination performance (Figure V in the online-only Data Supplement), with C-indices ranging from very low for SAH (0.57; 95% CI, 0.55–0.59) to relatively high for AAA (0.86; 95% CI, 0.85–0.88) in comparison with a conventional composite CVD model with C-index of 0.73 (95% CI, 0.72–0.73).

## Discussion

### Objectives Addressed, Summary of Main Findings

By linking EHRs from multiple sources we curated a cohort of nearly 2 million patients with >100 000 nonfatal and fatal CVD end points of 12 different types. We found that the majority of CVD first presentations are not MI or ischemic stroke but rather heart failure, angina, transient ischemic attack, and PAD. In our contemporary population-based cohort, we find that 51.3% of men and 41.2% of women experienced some form of CVD during their lifetime, with heart failure and stroke (primarily ischemic and NOS) becoming more common as the initial presentations in both men and women in later life. The variable associations of sex and age with different CVDs have important consequences for risk prediction.

### Importance of Studying First Manifestations of CVD

We compared the relative frequency of 12 of the most common CVDs affecting atherothrombotic processes in the coronary, cerebral, and peripheral circulations, aneurysms in the cerebral and peripheral circulations, and disorder of myocardial function and cardiac arrhythmia. This family of diseases is clinically relevant, because having one is strongly associated with the subsequent development of another and should initiate a range of secondary preventive interventions.^21^ Despite the insights to be gained from considering the first presentation among these diseases together, this first-lifetime-presentation approach has rarely been reported in the literature^22^ and has tended to exclude major diseases such as heart failure, been restricted to small cohorts, or reported in men only.^23–27^

### Innovative Role of Large-Scale Health Record Linkages

Through the use of linked EHRs, we were able to capture diseases first presenting in primary care and were not confined to hospitalized cases. Our cohort is population based, with >99% of the English population estimated to be registered with a family physician,^28^ unlike other recent large cohort studies, such as UK Biobank, with response rates <10%.^29^ The size of cohort— nearly 2 million people— possible with this EHR platform allows us to include serious but less commonly diagnosed events such as AAA and SAH, and to have sufficient events in women to study sex differences reliably. We were able to follow up actual events in a clinically meaningful 5-year time frame, similar to the time horizon of randomized, controlled trials. More broadly, we demonstrate the potential of linked EHR cohorts to complement bespoke, investigator-led cohorts. The UK Biobank,^30^ the Research Program for Genes Environment and Health in Kaiser,^31^ and precision medicine initiatives^32^ all place a major emphasis on specific disease types and follow-up through health records. Such large EHR cohorts further hold out the promise of lower cost to research funders for data collection, the intrinsic clinical relevance of real-world data, the opportunities to study diseases with higher specificity given the cohort sizes possible, and the prospect for researchers and clinicians to work across the boundaries that currently impede the translation of new discoveries into public health benefit.^33^

We expect the pattern of the age and sex associations we found with the CVD outcomes to apply to the broader UK population and other European populations free from symptomatic CVD. Our patients were drawn from >200 practices representative of the English population. Indeed, a recent article investigating similar questions in a smaller investigator-led Dutch cohort found broadly similar associations, albeit with fewer end points.^22^ Different cohorts, especially those with more people from differing ethnic groups or differing baseline risk profiles, may well present different associations.

### Validity of Risk Factor and Disease Measurements in EHRs

Although a principal strength of this study is the ability to resolve a wide range of CVDs in a large-scale cohort, the principal limitation is the possibility of errors in the individual EHR data sources.^34,35^ However, evidence for the validity of our risk factor and disease end points comes from several sources. First, in this population, using identical phenotypic definitions for these same 12 diseases, we have replicated anticipated risk factor – disease associations with systolic and diastolic blood pressure,^11^ type 2 diabetes mellitus,^15^ smoking,^10^ and socioeconomic deprivation.^12^ These findings support the prospective prognostic validity of both the risk factor and the disease measurements. Second, a recent systematic review of studies validating diagnoses in CPRD found a median positive predictive value of 88% across a wide range of diagnoses,^7^ whereas a separate systematic review found the accuracy of discharge coding in Hospital Episodes Statistics to be 83%.^35^ Third, the associations we found when considering events from all data sources (Figure V in the online-only Data Supplement) were consistent with those when excluding nonfatal cases or those from primary care. The doctors and coders responsible, and the information on which these diagnoses are based, differ for each data source (primary care, hospital, and death); it was reassuring that the associations were broadly similar. Finally, we^13^ and others^36^ have demonstrated the validity of using linked data for end point follow-up.

### Male Sex as a Risk Factor for Different CVDs

We demonstrate that male sex does not have a common underlying association on the incidence of different CVDs. Rather, the strength of this association is highly variable, ranging from protective for SAH; minor for transient ischemic attack, intracerebral hemorrhage, and unstable angina; moderate for stable angina, ischemic stroke, PAD, heart failure, and cardiac arrest/SCD; and strong for AAA, MI, and UCD. Additionally, we found that these associations change with age, with sex differences in proportion of initial presentation of MI and coronary death reducing with age, and with heart failure and stroke (ischemic and NOS) emerging as the most common initial presentations in both sexes. These findings suggest that stratifying patients into low-=, intermediate-, and high-risk groups based on their total and disease-specific risks,^37^ accompanied by the establishment of new cost-effective treatment thresholds,^38^ could improve risk management, particularly for diseases such as heart failure and stroke that affect high proportions of women but are undermanaged based on current clinical risk assessment.^39^

### Clinical Implications and Risk Prediction

Current risk algorithms in common use focus on CHD^40^ and CVD,^41^ as does the new American College of Cardiology/American Heart Association Guideline on the Assessment of Cardiovascular Risk,^42^ yet we show that chronic disease, such as heart failure and PAD, account for a substantial proportion of initial CVD presentations in contemporary practice. These diseases are associated with marked increased risk of subsequent events and death, yet have been excluded from many risk prediction algorithms. Given the recent decline in the incidence of acute events of MI and stroke, our findings raise the question of whether risk algorithms should take account of the current burden of CVDs and, in efforts to personalize cardiovascular risk, whether there is a need for risk algorithms tailored to account for specific diseases. For clinical use the latter would only have a role if decisions on prevention strategies were altered by using a more specific than a more generic risk prediction tool. Our post hoc analysis of the discrimination performance of risk prediction models using disease-specific age and sex coefficients supports the importance of having more tailored risk algorithms.

A more nuanced application of age and sex in the clinical setting that takes account of their heterogeneous associations with different CVDs is provided by the following example: A 69-year-old woman with untreated hypertension has a 20% 10-year general risk of CVD, fulfilling guideline criteria for primary prevention. With heart failure her most likely initial CVD presentation within that 10-year time frame (see Figure 1), a tailored blood pressure–lowering regime that excludes calcium antagonists would optimize CVD prevention because these drugs are relatively less effective at reducing risk of heart failure.^43^ At earlier ages, where CHD is the more common initial presentation, the choice of blood pressure–lowering medication is likely to make little difference to outcomes. This is just 1 example of the way in which understanding of the heterogeneity of risks associated with specific end points could lead to more personalized risk modification.

We also provide further evidence of the need to protect women against CVD with the same vigor as for men. The current strategy of evaluating and treating short-term risk of total CVD has the consequence that almost all men aged >70 should be on treatment, irrespective of their CVD risk factors. However, a wider group of people with high risk of specific CVDs could be targeted and treated earlier by increasing the sensitivity (by extending the time horizon to lifetime, as suggested by the Joint British Societies latest recommendations^44^) and specificity (by using more specific diagnoses) of risk predictions. Given that the majority of initial CVD presentations in our cohort were nonfatal (84% in men and 80% in women), such opportunities for earlier intervention via refinement of prediction tools should not be missed.

Furthermore, our findings have potentially important consequences for the accuracy of models used to predict CVD risk in clinical practice. We found large differences in the associations of different CVDs with age (from very weak with SAH to very strong with UCD, heart failure, stroke, and AAA) and male sex (from negative with SAH to very strong with AAA, nonfatal MI, and UCD). So far, most efforts to improve the prediction of CVD have focused on refining current models with new predictors. Although there are several models for specific CVDs (eg, heart failure,^45^ stroke^46^), current guidelines recommend assessment of total CVD risk to simplify clinical decision making.^21^ Here we show that this one-size-fits-all approach reduces the ability to discriminate between individuals with high and low risk of specific CVDs.

### Implications for Research

Our findings suggest that future research on the primary prevention of CVDs should take account of current patterns of disease presentation and redress the imbalance of previous literature that has focused extensively on heart attack and stroke. Our findings have implications for the design and interpretation of observational studies, randomized trials, and meta-analyses investigating the primary prevention of CVDs. Because the fundamental risk factors of age and sex have such heterogeneous associations with different CVDs, and most studies are only sufficiently powered to examine CVD aggregates, it is important to account for the relative proportion of each disease in the composite end point in meta-analysis. Despite an extensive literature on the underlying biological and behavioral pathways by which sex may influence aggregates of CVD and CHD, there is a lack of mechanism studies that investigate why sex has such heterogeneous associations on different CVDs.

### Limitations

Our study has important limitations. First, we were not able to resolve some disease subtypes, eg, systolic versus diastolic heart failure or ruptured versus nonruptured cases of AAA. We did find that the association of MI with male sex was more marked for ST-segment–elevation MI than non–ST-segment–elevation MI, suggesting an even greater degree of heterogeneity may be unmasked by investigating more specific diagnoses. Second, we did not evaluate common CVDs that are commonly asymptomatic such as atrial fibrillation. Third, EHRs contain limited covariates for explaining the heterogeneity in sex differences that we report. Fourth, there were 2 less well-specified diagnoses (CHD NOS and stroke NOS) which we were unable to resolve further, but which we included to ensure all potential initial presentations were taken into account. Stroke NOS is likely to be largely ischemic stroke, based on proportion of strokes that are ischemic^1^ and the behavior of this end point in modeling, indicating that we may have overestimated the association of ischemic stroke with male sex. We believe CHD NOS is a mixture of stable and unstable angina given the associations in this article and others, but are unable to substantiate this.

### Conclusion

In an era of modern primary prevention, CVDs commonly first present with heart failure, transient ischemic attack, stable angina, and PAD – diseases that have seldom been the focus of primary prevention studies. Predicting CVD risk should take account a wide range of CVDs, and the different association each has with age and sex, as well.

## Sources of Funding

This study was supported by the National Institute for Health Research (RP-PG-0407-10314), Wellcome Trust (WT 086091/Z/08/Z), and the Farr Institute of Health Informatics Research, funded by The Medical Research Council (K006584/1), in partnership with Arthritis Research UK, the British Heart Foundation, Cancer Research UK, the Economic and Social Research Council, the Engineering and Physical Sciences Research Council, the National Institute of Health Research, the National Institute for Social Care and Health Research (Welsh Assembly Government), the Chief Scientist Office (Scottish Government Health Directorates), and the Wellcome Trust. Dr George was funded by a National Institute for Health Research Doctoral Fellowship (DRF-2009-02-50). Dr Shah is supported by a Clinical Research Training Fellowship from the Wellcome Trust (0938/30/Z/10/Z). Dr Smeeth is supported by a Wellcome Trust Senior Research Fellowship in Clinical Science. Dr Timmis acknowledges support of St Bartholomew’s and the London Cardiovascular Biomedical Research Unit, funded by the National Institute for Health Research. This article presents independent research funded in part by the NIHR. The views expressed are those of the authors and not necessarily those of the NHS, the NIHR or the Department of Health. The authors declare that the funding sources had no role in the conduct, analysis, interpretation and writing of this manuscript.

## Disclosures

None.

## Supplementary Material

## CLINICAL PERSPECTIVES

The first lifetime presentation of cardiovascular disease in men and women in the 21st century is not currently well understood, with contemporary studies of sufficient size and clinical resolution to distinguish the most common cardiovascular diseases (CVDs) lacking. Traditional cohort studies have, to date, commonly focused on incident heart attack and stroke, but it is well recognized that both have been rapidly declining in incidence. Understanding how CVDs first present is important for developing primary prevention strategies that protect against specific phenotypes and against the wider cascade of other CVDs that often follow. Electronic health records based on usual clinical practice in unselected, contemporary populations provide an important opportunity to assess how CVD first presents in women and men across a wide range of 12 different diseases affecting the head, heart, abdominal, and peripheral circulations. In a study of 1.9 million adults, 114 859 people experienced an incident cardiovascular diagnosis, the majority (66%) of which were neither myocardial infarction nor ischemic stroke. Sex has differing associations with different CVDs, with implications for risk prediction and management strategies. Chronic disease, such as heart failure and peripheral arterial disease, account for a substantial proportion of initial lifetime CVD presentations, yet are been excluded from many risk prediction algorithms. Given the recent decline in the incidence of acute events, our findings emphasize the relevance of risk algorithms that take account of the current burden of CVDs.
