# Supplementary material for: How Does Cardiovascular Disease First Present in Women and Men? Incidence of 12 Cardiovascular Diseases in a Contemporary Cohort of 1 937 360 People
Source: Circulation. 2015 Oct 5;132(14):1320–8. doi: 10.1161/CIRCULATIONAHA.114.013797 (PMC4590518; doi:10.1161/CIRCULATIONAHA.114.013797)
Supplement: Supplementary file 1 [file cir-132-1320-s001.docx]

**Clinical Perspective - CIRCULATIONAHA/2014/013797R2**

The first lifetime presentation of cardiovascular disease in men and women in the 21^st^ century is not currently well understood, with contemporary studies of sufficient size and clinical resolution to distinguish the most common cardiovascular diseases (CVDs) lacking. Traditional cohort studies have to date commonly focussed on incident heart attack and stroke, but it is well recognised that both have been rapidly declining in incidence. Understanding how CVDs first present is important for developing primary prevention strategies that protect against specific phenotypes and against the wider cascade of other CVDs that often follows. Electronic health records based on usual clinical practice in unselected, contemporary populations provide an important opportunity to assess how CVD first presents in women and men across a wide range of 12 different diseases affecting the head, heart, abdominal and peripheral circulations. In a study of 1.9 million adults, 114,859 people experienced an incident cardiovascular diagnosis, the majority (66%) of which were neither myocardial infarction (MI) nor ischemic stroke. Sex has differing associations with different CVDs, with implications for risk prediction and management strategies. Chronic disease, such as heart failure and peripheral arterial disease, account for a substantial proportion of initial lifetime CVD presentations, yet are been excluded from many risk prediction algorithms. Given the recent decline in incidence of acute events, our findings emphasise the relevance of risk algorithms which take account of the current burden of CVDs.
