# Supplementary material for: How Does Cardiovascular Disease First Present in Women and Men? Incidence of 12 Cardiovascular Diseases in a Contemporary Cohort of 1 937 360 People
Source: Circulation. 2015 Oct 5;132(14):1320–8. doi: 10.1161/CIRCULATIONAHA.114.013797 (PMC4590518; doi:10.1161/CIRCULATIONAHA.114.013797)
Supplement: Supplementary file 2 [file cir-132-1320-s002.pdf]

SUPPLEMENTAL MATERIAL

Content

Supplemental methods

eText 1 Definition of risk factors

eText 2 Overview of codes and data sources used to define each cardiovascular endpoints

eText 3 Multiple imputation

Supplemental tables

eTable 1 Number and proportion of total events for wide range of cardiovascular diseases by gender and age group

Supplemental figures

eFigure 1 Study flow diagram

eFigure2 Hazard ratios of the association of baseline age with initial presentations of twelve different cardiovascular diseases, with age 40-49 years as reference

eFigure 3 Hazard ratios for men compared to women for initial presentations of twelve different cardiovascular diseases, adjusted for age, selected cardiovascular risk factors, and medications

eFigure 4 Age-adjusted hazard ratios for men compared to women for initial presentation of twelve cardiovascular diseases by different data sources

eFigure 5 The C-index of risk predictions based on CVD phenotype-specific models, adjusted for age & sex only

Supplemental References

Supplemental methods

eText1 Definition of risk factors

All risk factors were based on data recorded in CPRD during primary care consultations in the year prior to the index date, unless otherwise specified. Where multiple blood test results were recorded, the values from the test closest in time to the index date was used. Medications were deemed to be regular medications if patients had at least two repeat prescriptions, covering a two-month supply, in the year prior to the index date.

Patients' age was measured in years as at the index date. Socioeconomic status was based on an area measure of deprivation, the Index of Multiple Deprivation,[1] linked to their postcode (which was removed prior to receipt of the data). The most recent smoking record prior to the index date was used to classify patients as never, ex- or current smokers. Patients identified as current smokers with no smoking record within the three years before study entry were reclassified as having missing smoking data. Never smokers who had a previous record of smoking were reclassified as ex-smokers. Body mass index (kg/m2) was calculated using weight measurement closest in time to the index date. Patients were defined as diabetic if they ever had a diagnosis of diabetes or were receiving regular prescriptions for either insulin or metformin. Baseline SBP was based on readings taken during routine primary care consultations; where more than one measurement was taken on the same day, the average measurement was used. Both total cholesterol and high density lipoprotein were defined as the level in routine blood tests, in mmol/L.

Blood-pressure-lowering medications included in our definition are thiazide diuretics, beta-blockers, angiotensin converting enzyme-inhibitors, angiotensin receptor blockers, or calcium-channel blockers. Hormonal therapy (in women only) included combined oral contraceptives, progestogen-only oral contraceptives or hormone replacement therapy. Statins included atorvastatin, fluvastatin, pravastatin, rosuvastatin, or simvastatin.

Additionally the following co-variates were used for the multiple imputation:

- White cell count , haemoglobin, creatinine, alanine transferase from blood tests requested during routine clinical care;
- Regular prescriptions (repeat prescriptions covering period of at least two months) for aspirin; and
- Medical history of depression, cancer, renal disease, liver disease or chronic obstructive pulmonary disease.

eText 2 Overview of codes and data sources used to define each cardiovascular endpoints. (Further details given in the CALIBER data portal, www.caliberresearch.org/portal)

Endpoint	CPRD – Read codes	MINAP – specific disease registry	HES – OPCS 4 hospital procedures	HES – ICD 10 hospital diagnoses†	ONS – ICD 10 causes of death‡
**Stable angina**	G33..00: Stable Angina.<br>G33z.00: Angina pectoris NOS + 25 other codes for diagnosis of stable angina pectoris.<br>30 codes for evidence of coronary artery disease at angiography (CT,MR, invasive or not specified).<br>151 Read codes for evidence of myocardial ischaemia (Resting ECG, exercise ECG, stress echo, radioisotope scan).<br>Two or more successive prescriptions for anti-anginals.	nu	K40-K46: Coronary artery bypass graft.<br>K49,K50 and K75: Percutaneous coronary intervention, not within 30 days of an acute coronary syndrome.	I20: Stable angina pectoris excluding unstable angina (I20.0).	nu
**Unstable angina**	G311.13/G311100: Unstable angina.<br>G233200: Angina at rest.<br>G311400: Worsening angina + 13 other codes.	Discharge diagnosis of unstable angina, no raised ST elevation.<br>No raised troponin levels.	nu	I20.0: Unstable or worsening angina.<br>I24: Acute ischaemic heart disease.<br>I24.0: Coronary thrombosis not resulting in MI.	nu

Endpoint	CPRD – Read codes	MINAP – specific disease registry	HES – OPCS 4 hospital procedures	HES – ICD 10 hospital diagnoses†	ONS – ICD 10 causes of death‡
				I24.8: Other forms of ischaemic heart disease.<br>I24.9: Acute ischaemic heart disease, unspecified.	
**Coronary heart disease not otherwise specified**	G3…00: Ischaemic heart disease + 90 other codes including CHD NOS, chronic ischaemic heart disease, silent myocardial infarction.	nu	nu	CHD NOS, chronic ischaemic heart disease, silent MI (I25) excluding.I25.2, old MI.	nu
**Acute Myocardial Infarction (MI)**	G30X000: Acute ST segment elevation myocardial infarction.<br>G307100: Acute non-ST segment elevation myocardial infarction.<br>G30..14: Heart attack.<br>G30..15: MI Acute myocardial infarction + 60 other codes as Acute MI not otherwise specified.	MI with or without ST elevation based on initial electrocardiogram findings, raised troponins and clinical diagnosis.	nu	I21: Acute myocardial infarction.<br>I23: Current complications of acute MI.	nu

Endpoint	CPRD – Read codes	MINAP – specific disease registry	HES – OPCS 4 hospital procedures	HES – ICD 10 hospital diagnoses†	ONS – ICD 10 causes of death‡
**Unheralded coronary death**	Any CVD excluded.	Any CVD excluded.	Any CVD excluded.	Any CVD excluded.	I20: Angina Pectoris.<br>I21: Acute MI.<br>I22: Subsequent MI.<br>I23: Certain current complications following acute MI.<br>I24: Other acute ischaemic heart diseases.<br>I25: Chronic ischaemic heart disease.
**Heart failure**	G58..00: Heart Failure + 92 other Read codes for heart failure diagnosis.	nu	nu	I50: Heart failure.<br>I11.0: Hypertensive heart disease with (congestive) heart failure.<br>I13.0: Hypertensive heart and renal disease with (congestive) heart failure.<br>I13.2: Hypertensive heart and renal disease with both (congestive) heart failure and renal disease.	I50 Heart failure.<br>I11.0 Hypertensive heart disease with (congestive) heart failure.<br>I13.0: Hypertensive heart and renal disease with (congestive) heart failure.<br>I13.2: Hypertensive heart and renal disease with both (congestive) heart failure and renal disease

Endpoint	CPRD – Read codes	MINAP – specific disease registry	HES – OPCS 4 hospital procedures	HES – ICD 10 hospital diagnoses†	ONS – ICD 10 causes of death‡
**Ventricular arrhythmias, cardiac arrest and sudden cardiac death**	G574.00: Ventricular fibrillation and flutter.<br>G757.00: Cardiac arrest + 35 other Read codes for ventricular fibrillation, asystole, cardiac arrest, cardiac resuscitation, electro-mechanical dissociation.<br>G575100: Sudden cardiac death.	nu	X50: Implanted cardiac defibrillation device.<br>K59: Implantation, revision and renewal of cardiac defibrillator.	I46: Cardiac arrest.<br>I47.0: Re-entry ventricular arrhythmia.<br>I47.2: Ventricular tachycardia.	I46: Cardiac arrest.<br>I47.0: Re-entry ventricular arrhythmia.<br>I47.2: Ventricular tachycardia.
**Transient ischaemic attack**	Fyu5500: [X]Other transient cerebral ischaemic attacks + related symptoms + 5 other Read codes.	nu	nu	G458: Other transient cerebral ischaemic attacks and related syndromes.<br>G459: Transient cerebral ischaemic attack, unspecified.	nu
**Ischaemic stroke**	G64..11: CVA – cerebral artery occlusion, G64..13 Stroke due to cerebral arterial occlusion.<br>G6W..00: Cerebral infarction due to unspecified occlusion/stenosis of precerebral arteries.<br>G6X..00: Cerebral	nu	Stroke NOS with carotid endarterectomy or stenting within 90 days (OPCS codes L294, L295, L311, L314; Read codes 7A20300 + 4 others).	I63: Cerebral infarction.	I63: Cerebral infarction.

Endpoint	CPRD – Read codes	MINAP – specific disease registry	HES – OPCS 4 hospital procedures	HES – ICD 10 hospital diagnoses†	ONS – ICD 10 causes of death‡
	infarction due to unspecified occlusion/stenosis of cerebral arteries plus 8 other codes.				
**Subarachnoid haemorrhage**	G601.00:Subarachnoid haemorrhage from carotid siphon and bifurcation.<br>G602.00: Subarachnoid haemorrhage from middle cerebral artery.<br>G60X.00: Subarachnoid haemorrhage from intracranial artery, unspecified.	nu	nu	I60: Subarachnoid haemorrhage.	I60: Subarachnoid haemorrhage.
**Intracerebralhae morrhage**	Gyu6F00: [x] Intracerebral haemorrhage in hemisphere, unspecified + 16 other codes.	nu	nu	I61: Intracerebral haemorrhage.	I61: Intracerebral haemorrhage.
**Stroke not otherwise specified**	G66..11: Cerebrovascular accident unspecified + 14 other Read codes.	nu	U54.3: Delivery of rehabilitation for stroke.	I64: Stroke not specified as haemorrhage or infarction.<br>G463-G467: Stroke syndromes.	I64: Stroke not specified as haemorrhage or infarction.<br>I672: Cerebral atherosclerosis.<br>I679: Cerebrovascular disease, unspecified.

Endpoint	CPRD – Read codes	MINAP – specific disease registry	HES – OPCS 4 hospital procedures	HES – ICD 10 hospital diagnoses†	ONS – ICD 10 causes of death‡
**Peripheral arterial disease**	63 codes for lower limb peripheral arterial disease diagnosis (including diabetic PAD, gangrene, arterial thrombosis of the leg and intermittent claudication).<br>Evidence of atherosclerosis of iliac and lower limb arteries based on angiography or Dopplers.	nu	L50-L54: Bypass, reconstruction and other open operations on iliac artery.<br>L58-L60, L62: Bypass, reconstruction, transluminal operations or other open operations of femoral artery.<br>L65: Revision of reconstruction of artery.	I70.2: atherosclerosis of arteries of extremities.<br>I73.9: Peripheral vascular disease intermittent claudication<br>E10.05,E11-E14: Peripheral complications of diabetes including gangrene, insulin dependent diabetes mellitus, non-insulin-dependent diabetes mellitus, malnutrition-related diabetes mellitus, other specified diabetes mellitus, unspecified diabetes mellitus.	I70.2: Atherosclerosis of arteries of extremities.<br>I73.9: Peripheral vascular disease intermittent claudication.<br>Peripheral complications of diabetes including gangrene 0.5 suffix of E10: Insulin dependent diabetes mellitus, E11: Non-insulin-dependent diabetes mellitus, E12: Malnutrition-related diabetes mellitus, E13: Other specified diabetes mellitus, E14: Unspecified diabetes mellitus
**Abdominal aortic aneurysm**	G714.00: AAA without mention of rupture + 12 more codes for AAA diagnosis.<br>42 codes for AAA procedures.	nu	L16: Extra anatomic bypass of aorta.<br>L18-L23: Replacement of aneurysmal segment of aorta, bypass of segment of aorta, plastic repair of aorta.<br>L25-L28: Transluminal or endovascular insertion of stent on aneurysmal segment of	I71.3: Ruptured AAA.<br>171.4: AAA without rupture.<br>I71.5: Ruptured thoraco-abdominal aortic aneurysm.<br>I71.6: Thoracoabdominal aortic aneurysm without mention of rupture.<br>I71.8: Aortic aneurysm of	I71.3: Ruptured AAA.<br>I71.4: AAA without rupture.<br>I71.5: Ruptured thoraco-abdominal aortic aneurysm.<br>I71.6: Thoracoabdominal aortic aneurysm without mention of rupture.

Endpoint	CPRD – Read codes	MINAP – specific disease registry	HES – OPCS 4 hospital procedures	HES – ICD 10 hospital diagnoses†	ONS – ICD 10 causes of death‡
			aorta.	unspecified site, ruptured.<br><br>I71.9: Aortic aneurysm of unspecified site, without mention of rupture.	I71.8: Aortic aneurysm of unspecified site, ruptured.<br><br>I71.9: Aortic aneurysm of unspecified site, without mention of rupture.

Note: AAA, aortic abdominal aneurysm; CVD, cardiovascular disease; MI, myocardial infarction; NOS, not otherwise specified; nu = not used in definition; OPCS, Office of Population Censuses and Surveys Classification of Interventions and Procedures. †Primary cause of admission. ‡Underlying cause of death.

eText 3 Multiple imputation

Multiple imputation[2] was implemented using the *mice* algorithm in the statistical package R. Imputation models were estimated separately for men and women and included:

a) all the baseline covariates used in the main analysis (age, quadratic age, index of multiple deprivation, smoking, body mass index, diabetes, systolic blood pressure, total cholesterol, and HDL cholesterol);

b) prior (between 1 and 4 years before study entry) and post (between 0 and 1 year after study entry) averages of continuous main analysis covariates and other measurements not in the main analysis (white cell count, haemoglobin, creatinine, alanine transferase);

c) baseline medications (statins, blood pressure medications, aspirin, and oral contraceptives and hormone replacement therapy (in women only));

d) coexisting medical conditions (history of depression, cancer, renal disease, liver disease and chronic obstructive pulmonary disease);

e) the Nelson-Aalen hazard and the event status for each endpoint analysed in the data[3].

Non-normally distributed variables were log-transformed for imputation and exponentiated back to their original scale for analysis. Five multiply imputed datasets were generated, and Cox models fitted to each dataset. Coefficients were combined using Rubin's rules.
We checked whether the imputations were plausible by comparing plots of the distribution of observed and imputed values of all variables.

Supplemental Tables

eTable 1. Age and sex distribution of 60,155 events in men and 54,704 in women representing the first lifetime presentations of range of CVDs

Presentation	30-39 years		40-49 years		50-59 years		60-69 years		70-79 years		80+years		All ages	
	Men	Women	Men	Women	Men	Women	Men	Women	Men	Women	Men	Women	Men	Women
Myocardial infarction	746 (27.9)	174 (11.2)	1,942 (26.0)	441 (12.4)	3,038 (21.1)	883 (11.5)	2,536 (15.7)	1,392 (11.2)	1,765 (12.9)	1,708 (10.2)	584 (10.1)	1,030 (8.1)	10,611 (17.6)	5,628 (10.3)
Unstable angina	512 (19.2)	287 (18.5)	1,589 (21.3)	788 (22.2)	2,767 (19.2)	1,723 (22.5)	2,523 (15.6)	2,117 (17.0)	1,391 (10.2)	1,746 (10.4)	365 (6.3)	723 (5.7)	9,147 (15.2)	7,384 (13.5)
Stable angina	243 (9.1)	126 (8.1)	938 (12.6)	538 (15.2)	2,071 (14.4)	1,354 (17.7)	2,182 (13.5)	1,891 (15.2)	1,301 (9.5)	1,686 (10.1)	325 (5.6)	566 (4.4)	7,060 (11.7)	6,161 (11.3)
Coronary heart disease not otherwise specified	229 (8.6)	124 (8.0)	904 (12.1)	380 (10.7)	1,911 (13.3)	1,048 (13.7)	1,873 (11.6)	1,424 (11.5)	1,033 (7.6)	1,216 (7.3)	261 (4.5)	492 (3.9)	6,211 (10.3)	4,684 (8.6)
Peripheral arterial disease	224 (8.4)	197 (12.7)	695 (9.3)	394 (11.1)	1,671 (11.6)	856 (11.2)	1,795 (11.1)	1,380 (11.1)	1,339 (9.8)	1,682 (10.0)	444 (7.7)	842 (6.6)	6,168 (10.3)	5,351 (9.8)
Ischemic stroke	209 (7.8)	209 (13.5)	531 (7.1)	345 (9.7)	1,190 (8.3)	772 (10.1)	1,766 (10.9)	1,534 (12.3)	1,951 (14.3)	2,880 (17.2)	1,079 (18.6)	3,119 (24.5)	6,726 (11.2)	8,859 (16.2)
Cardiac arrest/ Sudden cardiac death	159 (6.0)	104 (6.7)	271 (3.6)	132 (3.7)	550 (3.8)	262 (3.4)	667 (4.1)	366 (2.9)	366 (2.7)	312 (1.9)	74 (1.3)	112 (0.9)	2,087 (3.5)	1,288 (2.4)
Transient ischemic attack	144 (5.4)	149 (9.6)	456 (6.1)	387 (10.9)	1,012 (7.0)	797 (10.4)	1,488 (9.2)	1,447 (11.6)	1,432 (10.5)	2,002 (11.9)	702 (12.1)	1,698 (13.3)	5,234 (8.7)	6,480 (11.8)
Heart failure	139 (5.2)	066 (4.2)	328 (4.4)	163 (4.6)	784 (5.4)	420 (5.5)	1,345 (8.3)	1,263 (10.2)	2,185 (16.0)	2,979 (17.8)	1,425 (24.6)	3,262 (25.6)	6,206 (10.3)	8,153 (14.9)
Unheralded coronary death	125 (4.7)	27 (1.7)	390 (5.2)	86 (2.4)	674 (4.7)	172 (2.2)	816 (5.0)	383 (3.1)	799 (5.8)	835 (5.0)	400 (6.9)	808 (6.3)	3,204 (5.3)	2,311 (4.2)
Stroke not otherwise specified	89 (3.3)	108 (7.0)	273 (3.7)	206 (5.8)	660 (4.6)	447 (5.8)	982 (6.1)	898 (7.2)	1,198 (8.8)	1,723 (10.3)	735 (12.7)	2,213 (17.4)	3,937 (6.5)	5,595 (10.2)
Hemorrhagic stroke	78 (2.9)	61 (3.9)	118 (1.6)	76 (2.1)	209 (1.5)	174 (2.3)	294 (1.8)	266 (2.1)	287 (2.1)	396 (2.4)	112 (1.9)	317 (2.5)	1,098 (1.8)	1,290 (2.4)
Subarachnoid hemorrhage	71 (2.7)	138 (8.9)	151 (2.0)	175 (4.9)	120 (0.8)	197 (2.6)	72 (0.4)	160 (1.3)	44 (0.3)	95 (0.6)	12 (0.2)	43 (0.3)	470 (0.8)	808 (1.5)
Abdominal aortic aneurysm	21 (0.8)	15 (1.0)	54 (0.7)	19 (0.5)	305 (2.1)	56 (0.7)	689 (4.3)	231 (1.9)	805 (5.9)	444 (2.6)	270 (4.7)	226 (1.8)	2,144 (3.6)	991 (1.8)

NB Columns in descending order of proportion by ordering in men 30-39 years.

Supplemental Figures

eFigure 1 Study flow diagram

eFigure 2 Hazard ratios of the association of baseline age with initial presentations of twelve different cardiovascular diseases, with age 40-49 years as reference

eFigure 3 Hazard ratios for men compared to women for initial presentations of twelve different cardiovascular diseases, adjusted for age, selected cardiovascular risk factors, and medications

eFigure 4 Age-adjusted hazard ratios for men compared to women for initial presentation of twelve cardiovascular diseases by different data sources

eFigure 5 The C-index of risk predictions based on CVD phenotype-specific models, adjusted for age & sex only

Supplemental References

1. Noble M, McLennan D, Wilkinson K, Whitworth A, Exley S, Barnes H, Dibben C, 2007, *The English Indices of Deprivation.* Wetherby: Department of Communities and Local Government. 2007.

2. Rubin DB. *Multiple Imputation for Nonresponse in Surveys*. *New York*: John Wiley and Sons. 1987.

3. White IR, Royston P. Imputing missing covariate values for the Cox model. *Stat Med*. 2009;28:1982–1998.

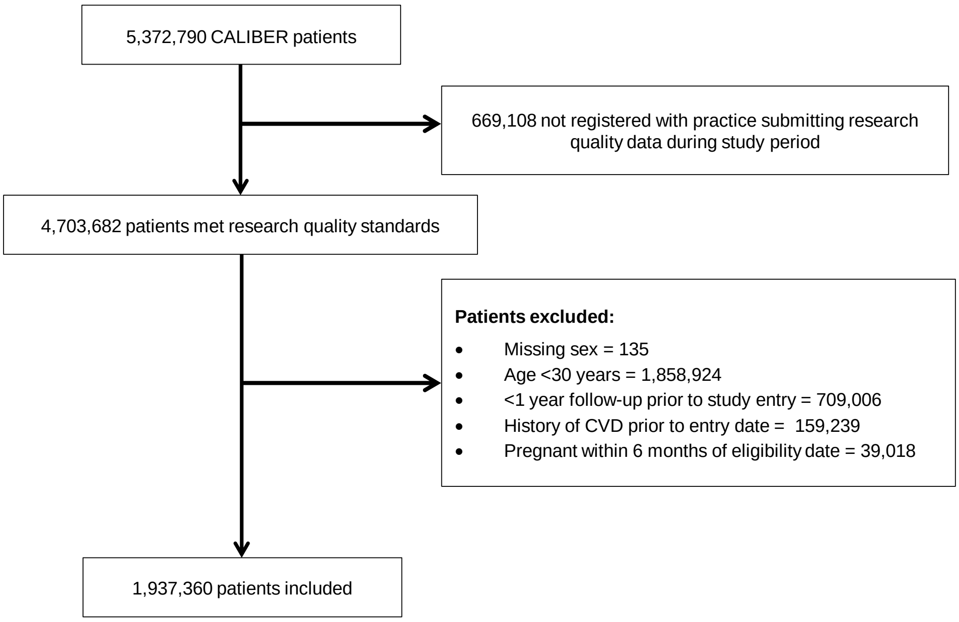
5,372,790 CALIBER patients
669,108 not registered with practice submitting research quality data during study period
4,703,682 patients met research quality standards
Patients excluded:
Missing sex = 135
Age <30 years = 1,858,924
<1 year follow-up prior to study entry = 709,006
History of CVD prior to entry date = 159,239
Pregnant within 6 months of eligibility date = 39,018
1,937,360 patients included

eFigure 1

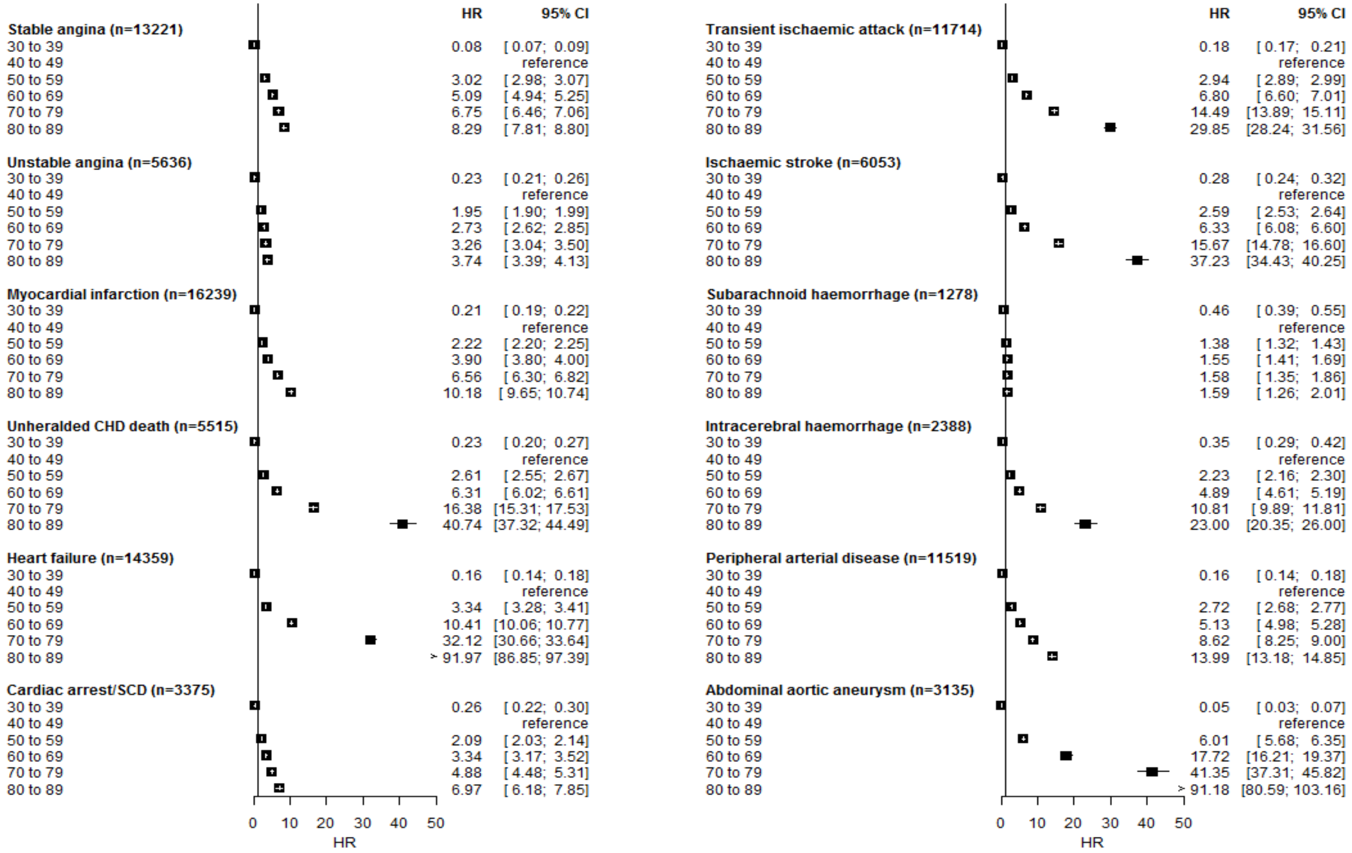

HRs comparing age groups to reference age group (40-49 years), adjusted for sex and stratified by primary care practice. CHD indicates coronary heart disease; SCD, sudden coronary death.

eFigure 2

eFigure 3

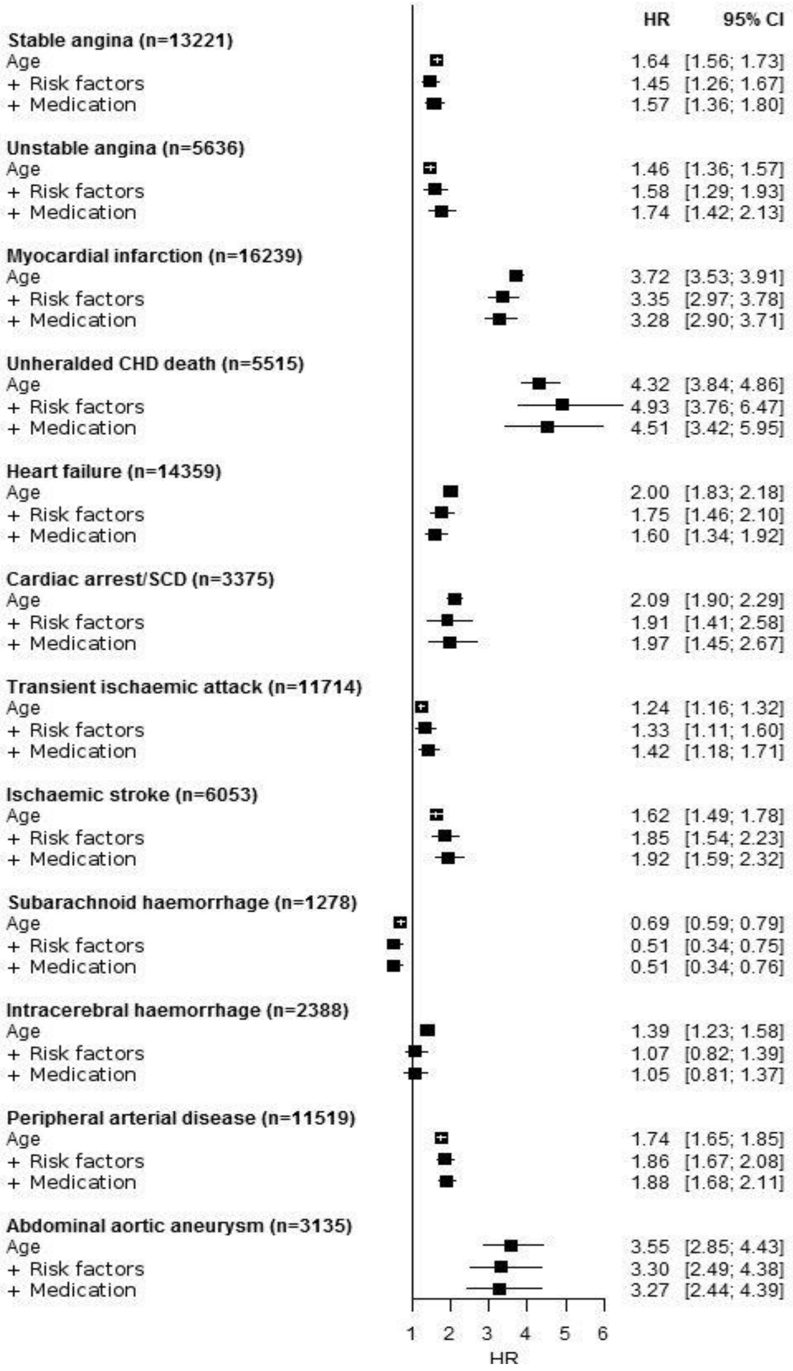

HRs adjusted for age, risk factors and medications, with missing data handled using multiple imputation. Adjustment for risk factors included smoking status, body mass index, systolic blood pressure, total and high-density lipoprotein cholesterol, diabetes mellitus and social economic status. Adjustment for medications included statins, blood pressure medications (includes thiazides, beta-blockers, ARB/ACE inhibitors) and, in women only, oral contraceptives or hormone replacement therapy. CHD indicates coronary heart disease; SCD, sudden cardiac death.

eFigure 4

	Events	HR	95% CI
**Stable angina**			
Primary care, secondary care & mortality	13221	1.64	[1.56; 1.73]
Secondary care & mortality	1277	2.91	[2.49; 3.40]
**Unstable angina**			
Primary care, secondary care & mortality	5636	1.46	[1.36; 1.57]
Secondary care & mortality	9765	1.51	[1.43; 1.59]
**CHD NOS**			
Primary care, secondary care & mortality	10895	2.03	[1.92; 2.15]
Secondary care & mortality	10143	3.04	[2.86; 3.22]
**Myocardial infarction**			
Primary care, secondary care & mortality	16239	3.72	[3.53; 3.91]
Secondary care & mortality	15359	3.81	[3.61; 4.02]
**Unheralded CHD death**			
Primary care, secondary care & mortality	5515	4.32	[3.84; 4.86]
Secondary care & mortality	9014	4.33	[3.92; 4.77]
Mortality	14882	4.31	[3.96; 4.70]
**Heart failure**			
Primary care, secondary care & mortality	14359	2.00	[1.83; 2.18]
Secondary care & mortality	7435	1.99	[1.77; 2.24]
Mortality	2013	2.34	[1.71; 3.20]
**Cardiac arrest/sudden cardiac death**			
Primary care, secondary care & mortality	3375	2.09	[1.90; 2.29]
Secondary care & mortality	3659	2.23	[2.03; 2.44]
**Transient ischaemic attack**			
Primary care, secondary care & mortality	11714	1.24	[1.16; 1.32]
Secondary care & mortality	3689	1.19	[1.08; 1.31]
**Ischaemic stroke**			
Primary care, secondary care & mortality	6053	1.62	[1.49; 1.78]
Secondary care & mortality	8124	1.71	[1.58; 1.85]
Mortality	971	1.96	[1.31; 2.93]
**Subarachnoid haemorrhage**			
Primary care, secondary care & mortality	1278	0.69	[0.59; 0.79]
Secondary care & mortality	1603	0.67	[0.59; 0.76]
Mortality	616	0.60	[0.48; 0.74]
**Intracerebral haemorrhage**			
Primary care, secondary care & mortality	2388	1.39	[1.23; 1.58]
Secondary care & mortality	2634	1.48	[1.31; 1.67]
Mortality	1344	1.48	[1.22; 1.81]
**Stroke NOS**			
Primary care, secondary care & mortality	9532	1.37	[1.26; 1.49]
Secondary care & mortality	6857	1.56	[1.37; 1.78]
Mortality	5388	2.56	[1.84; 3.56]
**Peripheral arterial disease**			
Primary care, secondary care & mortality	11519	1.74	[1.65; 1.85]
Secondary care & mortality	3755	2.22	[2.02; 2.45]
Mortality	341	2.56	[0.95; 6.93]
**Abdominal aortic aneurysm**			
Primary care, secondary care & mortality	3135	3.55	[2.85; 4.43]
Secondary care & mortality	2706	5.18	[3.94; 6.81]
Mortality	1901	4.22	[3.03; 5.88]
**Other death**			
Primary care, secondary care & mortality	60897	1.27	[1.24; 1.31]
Secondary care & mortality	91273	1.35	[1.32; 1.38]
Mortality	109438	1.36	[1.33; 1.38]

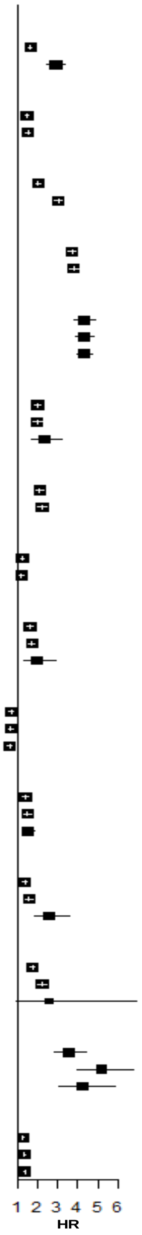

HRs comparing men to women by source of endpoint data, adjusted for age and stratified by primary care practice. CHD indicates coronary heart disease; NOS, not otherwise specified.

eFigure 5

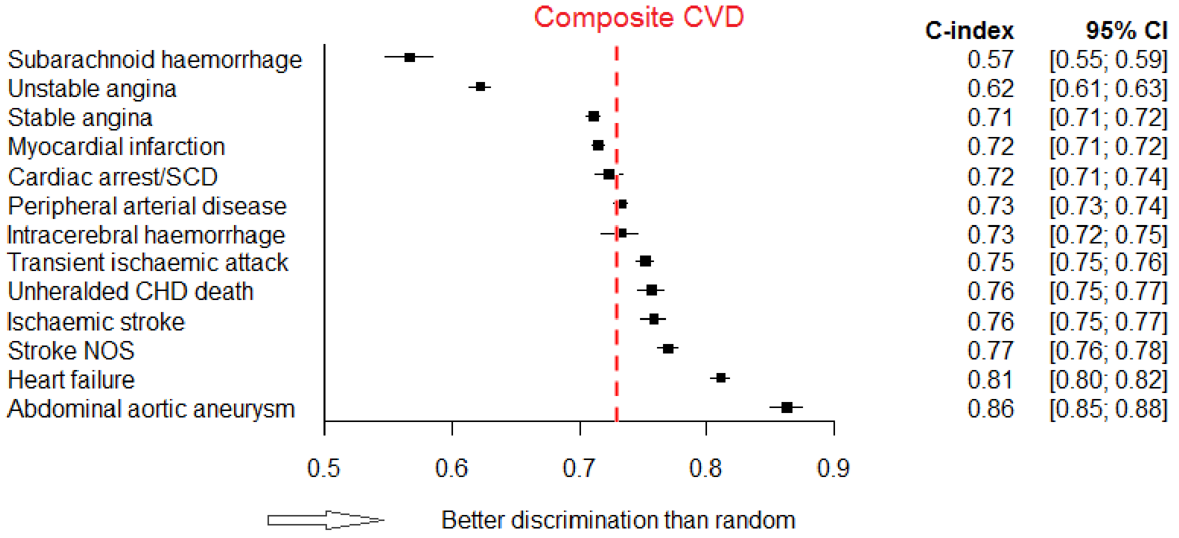

Data restricted to baseline age 40 to 74 (N= 1,004,190). Composite CVD (red dashed line), C-index 0.73 (95% CI, 0.72, 0.73). SCD indicates sudden cardiac death; CHD, coronary heart disease, NOS, not otherwise specified
